# Browsers or Grazers? New Insights into Feral Burro Diet Using a Non-Invasive Sampling and Plant DNA Metabarcoding Approach

**DOI:** 10.3390/ani13162683

**Published:** 2023-08-21

**Authors:** Saeideh Esmaeili, Sarah R. B. King, Kathryn A. Schoenecker

**Affiliations:** 1 Natural Resource Ecology Laboratory, Colorado State University, and in Cooperation with USGS Fort Collins Science Center, Fort Collins, CO 80523, USA; sarah.king@colostate.edu; 2 U.S. Geological Survey, Fort Collins Science Center, Fort Collins, CO 80526, USA; schoeneckerk@usgs.gov; 3 Ecosystem Science and Sustainability, Colorado State University, Fort Collins, CO 80523, USA

**Keywords:** Bureau of Land Management, diet, donkey, *Equus asinus*, feral burro, Lake Pleasant HMA, Sinbad HMA

## Abstract

**Simple Summary:**

By selecting certain plants for consumption, ungulates (hoofed mammals) shape ecosystems and influence which plant species are present in their habitats. We investigated the summer diets of non-native feral burros in two ecosystems: a subtropical Sonoran Desert in Arizona and a temperate juniper shrubland in Utah, the United States. In June and July of 2019, we gathered 50 fecal samples from both locations and analyzed plant DNA in the samples to identify which plants the burros were eating. Our findings revealed that during our summer sampling period, the burros in the Sonoran Desert predominantly consumed woody browse, whereas the burros in the juniper woodland consumed a wide range of flowering herbaceous plants (forbs) and grasses. The burros in the temperate system had to consume a more diverse diet to meet their nutritional needs, while the burros in the Sonoran Desert could rely on two major forage species, mesquite and grasses from the *Poaceae* family; as a result, their diet had a lower degree of diversity. Feral burros are descended from the African wild ass and exhibit a similar mixed feeding strategy to their ancestors in which they can adapt their diet in different ecosystems to meet their nutritional requirements.

**Abstract:**

Ungulates play a large role in shaping ecosystems and communities by influencing plant composition, structure, and productivity. We investigated the summer diets of feral burros in two ecosystems in which they are found in the United States: a subtropical desert in Arizona and a temperate juniper shrubland in Utah. Between 24 June and 16 July of 2019, we gathered 50 burro fecal samples from each location and used plant DNA metabarcoding to determine the burros’ diets. We found that during our sampling period the burros in the Sonoran Desert consumed a higher proportion of woody browse and had a narrower dietary niche breadth and lower degree of diet diversity compared to the burros in the juniper shrubland ecosystem, where the burros consumed higher proportions of graminoids and forbs and had a higher diet diversity index and broader dietary niche breadth. The burros in the Sonoran Desert relied primarily on *Prosopis* spp. (mesquite) and *Poaceae* grasses, whereas the burros in the juniper shrubland relied on a wider variety of forb and grass species, likely due to the greater variability in the forage species temporally and spatially available in that temperate ecosystem. We found that feral burros are highly adaptable with respect to diet and appear to be employing a mixed feeding strategy, similar to their ancestor, the African wild ass, to meet their nutritional needs in whichever ecosystem they are found.

## 1. Introduction

Large herbivores play a significant role in shaping ecosystems by influencing the composition, structure, diversity, and productivity of plant communities [1,2,3,4]. Their grazing pressure and selectivity trigger facilitative or competitive interactions that modify the available forage for other wild and domestic herbivores in the community, thereby defining the functioning of the entire ecosystem [1,5,6]. The critical role herbivores play in shaping ecosystems by influencing plant communities and maintaining open landscapes has been widely recognized and applied for ecosystem management [7,8]. To understand the overall effect of herbivores on the ecosystem and other species, it is essential to identify the types and relative abundances of forages consumed by these animals, thus gaining insights into their ecological roles and dietary niche within the community [9].

Feral burros (*Equus asinus*), also referred to as donkeys, originated from the African wild ass (*E. africanus*) and were introduced to North America as pack animals by Spanish explorers in the 1530s [10]. Feral populations of burros became established mostly in the southwestern United States after their traditional use declined during the industrial age. Currently, the Bureau of Land Management (BLM) and the U.S. Forest Service manage feral burros and feral horses (*E. caballus*) in specific management areas. Despite their long-term persistence, the ecological roles feral burros play in shaping western ecosystems have been largely understudied compared to native ungulates [11]. Due to their African wild ass ancestry and hindgut fermentation digestion, burros are adapted to arid and low-productivity environments, enabling them to tolerate adverse nutritional conditions and consume low-quality plants efficiently [12,13]. Their physiological and behavioral adaptations to arid environments allow burros to utilize forages that may not be accessible to ruminant species, thereby allowing them a broader diet breadth [13,14]. Understanding the dietary preferences and habits of feral burros provides insights into their effects on plant communities, interactions with other animals, and a more comprehensive understanding of their ecological role within the ecosystem. 

Feral burros exhibit browsing behavior, consuming a higher proportion of forbs (flowering herbaceous plants) and browse (shrubs and woody plants) compared to horses, which are primarily grazers ([15]; see Table 1 for a review of burro diets). This dietary difference is attributed to the physiological and cranial musculoskeletal adaptations of burros, which enable them to utilize different parts of shrubs [12,16]. This unique feature has been utilized to control and reduce shrub encroachment where it is considered problematic [17]. Additionally, graminoids can play a significant role in burro diets (Table 1 and the references therein). The wide dietary niche of feral burros positions them as strong potential competitors for both wildlife and livestock ([18,19]; however, see [20]). Therefore, understanding their diets in different ecosystems is important for ecology and the management of landscapes with burros.

The existing literature on the diet of feral burros has predominantly relied on direct observations, microhistological methods, and stomach content analyses (summarized in Table 1). Direct observation can be time-consuming and prone to bias, particularly when plant species are not easily recognizable from a distance [21]. Microhistological results are often influenced by the differential digestibility of plant species, leading to underestimations of more completely digested plants, such as forbs [21,22]. The use of stomach content analysis is rare because it is an invasive procedure requiring restrained or culled animals [21]. In recent years, DNA metabarcoding has emerged as a powerful tool for identifying the diets of various animals from carnivores to herbivores [23,24]. This approach involves amplifying and sequencing specific regions of DNA, such as chloroplast or mitochondrial DNA, extracted from animal fecal samples. By comparing the obtained DNA sequences with those in a plant reference database, it is possible to accurately identify the plant species consumed by animals [25]. Plant DNA metabarcoding has been successfully employed to estimate plant species in the diets of other equid species, providing a reliable and useful estimation of their dietary compositions [22,23].

Feral horses tend to receive more attention than feral burros, resulting in a lack of information on burro ecology [11]. In the United States there are approximately 14,000 burros on BLM-managed lands [26], with over 2 million feral burros estimated to be in Australia [27]. They exist in a variety of ecosystems across these continents and others, and there is therefore a need to understand the effect of burros on the landscape across multiple habitat types. The primary objective of this study was to investigate the diet compositions of feral burros in two distinct ecosystems (a subtropical desert and a temperate juniper shrubland) where feral burros are found in the United States. We aimed to advance our understanding of burro diet and ecology through the application of a plant DNA metabarcoding approach. By employing this technique, we sought to refine and expand existing information about feral burro diets, thus contributing to the existing body of knowledge on the ecology of the species.

**Table 1:** Summary of plant forms comprising the annual and seasonal diets of feral burros and domestic donkeys reported in the literature between 1973 and 2014. For studies in which the sum of the plant forms in the diet does not equal 100%, a portion of the diet was not assigned to the defined categories.

Time of Year	Method of Analysis	Location	Reference	% Graminoids	% Forbs	% Shrub/ Tree
** Spring **						
March	Microhistology	California, USA	Woodward and Ohmart 1976 [28]	2.20	77.40	19.50
April	Stomach contents	California, USA	California Fish and Game 1966 (in [16])	1.00	98.00	1.00
April	Microhistology	California, USA	Woodward and Ohmart 1976 [28]	7.70	58.20	34.10
May	Microhistology	California, USA	Woodward and Ohmart 1976 [28]	0.20	51.90	38.10
April–June	Microhistology	California, USA	Marshal et al., 2012 [29]	15.10	15.90	65.40
Spring	Microhistology	Arizona, USA	Seegmiller and Ohmart 1981 [10]	30.10	34.50	30.40
			** Mean **	** 9.38 **	** 55.98 **	** 31.42 **
			** SD **	** 11.58 **	** 29.37 **	** 21.33 **
** Summer **						
June	Microhistology	California, USA	Woodward and Ohmart 1976 [28]	0.00	37.20	58.00
July	Microhistology	California, USA	Woodward and Ohmart 1976 [28]	2.00	12.10	82.30
July	Stomach contents	Arizona, USA	Jordan and Colton 1979 [30]	47.80	17.40	31.80
August	Microhistology	California, USA	Woodward and Ohmart 1976 [28]	2.40	13.90	78.80
August	Stomach contents	Arizona, USA	Jordan and Colton 1979 [30]	34.40	15.20	48.70
August	Microhistology	Arizona, USA	Potter and Hansen 1979 [31]	66.00	16.00	11.00
July–September	Microhistology	California, USA	Marshal et al., 2012 [29]	11.80	13.50	72.30
Summer	Microhistology	Arizona, USA	Seegmiller and Ohmart 1981 [10]	33.10	11.20	48.60
Summer	Direct observation	Belgium	Cosyns et al., 2001 [32]	60.60	10.40	29.00
Summer	Direct observation	India	Mishra et al., 2004 [33]	61.00	30.00	9.00
Summer	Microhistology	Argentina	Reus et al., 2014 [34]	56.76	0.50	32.94
			** Mean **	** 34.17 **	** 16.13 **	** 45.68 **
			** SD **	** 26.15 **	** 9.84 **	** 25.49 **
** Fall **						
September	Microhistology	California, USA	Woodward and Ohmart 1976 [28]	2.30	8.40	83.80
September	Stomach contents	Arizona, USA	Jordan and Colton 1979 [30]	23.30	10.10	64.20
October	Microhistology	California, USA	Woodward and Ohmart 1976 [28]	12.60	8.00	74.00
October–December	Microhistology	California, USA	Marshal et al., 2012 [29]	13.60	19.90	65.40
Fall	Direct observation	Belgium	Cosyns et al., 2001 [32]	79.50	7.40	13.10
			** Mean **	** 26.26 **	** 10.76 **	** 60.10 **
			** SD **	** 30.68 **	** 5.21 **	** 27.43 **
** Winter **						
November	Microhistology	California, USA	Woodward and Ohmart 1976 [28]	2.90	10.90	82.90
December	Microhistology	California, USA	Woodward and Ohmart 1976 [28]	14.30	11.20	73.10
January	Microhistology	California, USA	Woodward and Ohmart 1976 [28]	0.00	22.70	73.80
February	Microhistology	California, USA	Woodward and Ohmart 1976 [28]	1.20	46.90	36.00
January–March	Microhistology	California, USA	Marshal et al., 2012 [29]	15.50	13.10	69.20
Winter	Microhistology	Arizona, USA	Seegmiller and Ohmart 1981 [10]	1.80	56.50	39.60
Winter	Direct observation	Belgium	Cosyns et al., 2001 [32]	86.00	6.60	7.40
Winter	Direct observation	India	Mishra et al., 2004 [33]	86.00	14.00	0.00
Winter	Microhistology	Argentina	Reus et al., 2014 [34]	33.15	2.19	40.53
			** Mean **	** 26.76 **	** 20.45 **	** 46.95 **
			** SD **	** 35.18 **	** 18.71 **	** 29.95 **
** Annual **						
Annual	Stomach contents	California, USA	Browning 1960 [35]	10.00	39.00	51.00
Annual	Microhistology	Arizona, USA	Hansen and Martin 1973 [36]	68.60	9.00	23.00
Annual	Microhistology	California, USA	Woodward and Ohmart 1976 [28]	3.90	30.10	61.10
Annual	Microhistology	California, USA	Douglas and Hiatt 1987 [37]	48.00	19.00	25.00
Annual	Microhistology	Arizona, USA	Seegmiller and Ohmart 1981 [10]	22.00	33.00	40.00
Annual	Microhistology	California, USA	Ginnett 1982 [38]	41.00	3.00	48.00
Annual	Direct observation	Belgium	Cosyns et al., 2001 [32]	69.00	13.00	18.00
Annual	Direct observation	Belgium	Lamoot et al., 2005 [39]	80.00	10.00	10.00
Annual	Literature review	California, USA	Abella 2008 [40]	30.00	26.00	38.00
Annual	Microhistology	Argentina	Borgnia et al., 2008 [41]	88.30	2.20	6.90
			** Mean **	** 46.08 **	** 18.43 **	** 32.10 **
			**SD**	**29.65**	**13.00**	**18.27**

## 2. Materials and Methods

### 2.1. Study Area

We conducted our study in two separate burro populations in the United States: the Lake Pleasant Herd Management Area (HMA), Arizona, population and the Sinbad HMA, Utah, population. There were approximately 300 burros in the Lake Pleasant HMA and 130 burros in the Sinbad HMA at the time of our study [42]. The Lake Pleasant HMA is located within the Sonoran Desert, covering 419 km^2^, although burros use areas outside of the HMA as well [42]. The average (mean ± SD) monthly temperature and precipitation were 21.0 ± 8.5 °C and 39.1 ± 41.5 mm in 2019, respectively (with December and February being the wettest months and June and October being the driest months; PRISM Time Series Data: January–December 2019). The vegetation communities mainly consist of succulents such as saguaro (*Carnegiea gigantea*), prickly pear (*Opuntia phaeacantha*), cholla (*Cylindropuntia* spp.), and ocotillo (*Fouquieria splendens*). Woody plants in the area include acacia (*Senegalia* spp.), creosote bush (*Larrea tridentata*), tamarisk (*Tamarix* spp.), and leguminous trees such as palo verde (*Parkinsonia* spp.), and mesquite (*Prosopis* spp.). Arizona cottontop (*Digitaria californica*), curly mesquite grass (*Hilaria belangeri*), big galleta (*Pleuraphis rigida*), Bigelow bluegrass (*Poa bigelovii*), little barley (*Hordeum pusillum*), sixweeks fescue (*Vulpia octoflora*), grama (*Bouteloua* spp.), and panic grasses (e.g., *Brachiaria arizonica* and *Panicum hirticaule*) comprise herbaceous vegetation in the understory [43,44]. 

The Sinbad HMA is located on the San Rafael Swell in central Utah and covers 402 km^2^ of canyonlands and open grasslands; similar to Lake Pleasant, burros are also found outside HMA boundaries [42]. In 2019, the average monthly temperature and precipitation were 9.9 ± 10.1 °C and 23.6 ± 17.4 mm, respectively (with May being the wettest month and October being the driest month; PRISM Time Series Data: January–December 2019). The vegetation communities comprise mainly juniper (*Juniperus* spp.) shrubland with open meadow grasslands. Woody vegetation at the site includes juniper and Piñon pine (*Pinus edulis*), with shrubs such as sagebrush (*Artemisia* spp.), yellow rabbitbrush (*Chrysothmanus viscidiflorus*), ephedra (*Ephedra torreyana*), and yucca (*Yucca harrimaniae*). Herbaceous plants including needle-and-thread grass (*Hesperostipa comata*), Indian ricegrass (*Oryzopsis hymenoides*), James’ galleta (*Hilaria jamesii*), and *Astragalus* spp. [45,46] comprise grassland meadow and shrub understory vegetation.

### 2.2. Fecal Sample Collection

**Figure 1:**
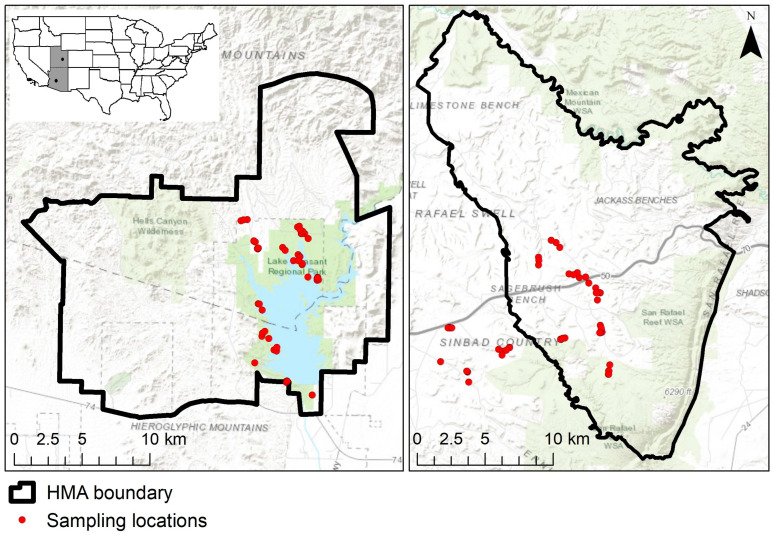
Location of fecal samples collected at the Lake Pleasant Herd Management Area (HMA), Arizona (left, N = 50), and Sinbad HMA, Utah (right, N = 50), over one month, 24 June to 16 July, in 2019. The inset shows the locations of the two study areas in the United States.

We collected 50 fresh fecal samples randomly from across each HMA while field monitoring the burros [47] between 24 June 2019 and 16 July 2019 (Figure 1), using the same method at both sites. We categorized the fecal piles as “fresh”, using previously published descriptions and guidelines [47]; the burro individual depositing the sample was not known in most cases. When we encountered a fresh fecal pile in areas commonly used by burros, we removed one fecal bolus from the pile using nitrile gloves or a tongue depressor and placed it into a paper bag. The samples in paper bags were placed within a large cotton bag and suspended in a hot, dry location (field trailers) to air dry.

### 2.3. Plant DNA Metabarcoding

We rehydrated the dried samples in ethanol and sent them to Jonah Ventures Laboratory (https://jonahventures.com; accessed on 12 February 2023) for analysis using DNA metabarcoding with chloroplast gene trnL primers [25], as described in [22,48] and the references therein. Sequencing success and read quality were assessed using FastQC v0.11.8. The reads were demultiplexed using Illumina-utils v2.6 (iu-demultiplex) with default settings. Subsequently, the sequences of each sample were merged using the -fastq_mergepairs option in Usearch v11.0.667 [49]. The forward primer (5′- CGAAATCGGTAGACGCTACG-3′) and reverse primer (5′- CCATTGAGTCTCTGCACCTATC-3′) were removed using Cutadapt v1.18 [50]. Additionally, Cutadapt was used to discard sequences below 108 bp in length. To filter out low-quality reads, the expected error filtering method, implemented in Usearch with a max_ee = 0.5, was employed [51]. Instead of performing operational taxonomic unit (OTU) clustering, the unoise3 algorithm was utilized with an alpha value of 5 to remove reads affected by sequencing and PCR errors [52]. This denoising step was applied to each individual sample, resulting in the compilation of exact sequence variants (ESV) in an ESV table, including sequences and read counts for each sample. Using usearch_global, taxonomy assignment was performed for each ESV via mapping against the GenBank reference data [53] and Jonah Ventures voucher sequence records. Mapping accuracy was ensured by setting --maxaccepts 0 and --maxrejects 0. A consensus taxonomy was generated from the hit tables by considering 100% matches initially and gradually reducing the match threshold in 1% steps until hits were available for each ESV. For ESVs with multiple matching taxa, the taxonomy present in at least 90% of the hits was reported, or “NA” was reported if no consensus was reached. To minimize errors stemming from misidentified taxa, the match threshold was increased to 2% if matches of 97% or higher were detected, and no family-level taxonomy was assigned in such cases. 

We used exact sequence variants (ESVs, i.e., unique taxonomic units derived from the DNA sequence) representing 95% of all unique sequence reads at each study area for the statistical analyses. This percentage, 95% of all reads, comprised 170 ESVs in the Lake Pleasant HMA and 220 ESVs in the Sinbad HMA, with no single ESV comprising more than 5% of any sample. We standardized the relative abundance of each sample so the sum of all reads of the top 170 and 220 ESVs (at Lake Pleasant and Sinbad HMAs, respectively) totaled 100%. To describe the taxonomic composition of the diet, we matched ESVs to the representative taxa, using a match criteria threshold of >90% similarity to the reference sequences [54,55]. If the representative species assigned to each ESV did not exist in our study areas, we assigned another species of the same genus to the ESV based on the list of plant species in each area (Lake Pleasant [56]; Sinbad [45,46,47,48,49,50,51,52,53,54,55,56,57]). Exact sequence variants that were identified at only the family level or their identified genera were not present in the study area and were only included in family-level analyses. If the representative family did not exist in the study area or the ESV was not identified at the family level, we removed it from analysis and standardized the relative abundances of remaining ESVs so the sum of all reads totaled 100% [48,55]. ESVs that matched similar genera were combined and labeled as operational taxonomic units (OTUs). For example, multiple ESVs that matched “*Poa* spp.” were combined into one unique OTU for this genus at each study area [22,55]. 

To investigate the plant form composition of burro diets, we categorized each ESV or OTU into four groups: forbs, graminoids, woody plants (including shrubs and trees), and “others” (including moss and vine), using the U.S. Department of Agriculture online plants database (https://plants.usda.gov; accessed 9 March 2023) to categorize representative species into plant form groups. For family-level ESVs, we made the selection based on the plant forms represented in each family; if the entire family consisted of one plant form, such as *Poaceae*, which are only graminoids, we assigned that family it’s single plant form. If the family encompassed multiple plant forms (such as *Asteraceae*), we labeled the plant form of the family “unknown”. We combined OTUs and ESVs by plant form to calculate their percentages in the diets of the burros in the two study areas.

### 2.4. Statistical Analyses

We processed data in Microsoft Excel and conducted all statistical analyses using R software [58]. To quantify diet composition, we divided each unique sequence read by the total sequence read for each sample to transform the sequence read count data into the relative read abundance. We used the Shannon diversity index (H), which represents both the abundance and evenness of the taxa in the diet, to calculate diet diversity for each burro population (using the vegan package in R [59]). We calculated the average ESV and OTU richness per sample to determine the dietary niche breadth at each study area. We performed the analyses on two different scales: ESV and OTU. The ESV scale allowed us to provide information at a taxonomy-free level, while the OTU scale offered information at the identified taxonomic level. Because the reference databases are often incomplete and prone to improve over time, taxonomy-free analyses retain information on sequences belonging to the same species which were previously unassigned and have poorly identified ESVs [55,60]. We compared diet diversity and dietary niche breadth between the two study populations using non-parametric Wilcoxon signed rank tests because our data did not meet the assumptions for a parametric test. 

## 3. Results

We identified 944 unique sequence reads (32.12 ± 10.06 ESVs per sample) in Lake Pleasant and 1094 unique reads (27.84 ± 9.63 ESVs per sample) in Sinbad. On average, we recorded 9.12 ± 3.43 and 12.20 ± 4.53 ESVs per sample in 95% of the reads in the Lake Pleasant and Sinbad HMAs, respectively. At Lake Pleasant, 8.6% of the top 95% ESVs could not be assigned to any family and were removed from analyses, and at Sinbad, 5.5% of the top 95% ESVs could not be assigned to any family and were removed from analyses. Since we standardized the relative abundances of the samples so the sum of all reads of the remaining ESVs equaled 100%, subsequent results are based on 148 ESVs at Lake Pleasant and 202 ESVs at Sinbad. At the genus level, we identified 56 and 52 OTUs at Lake Pleasant and Sinbad, respectively. On ESV scales, the Sinbad population had a more diverse diet than the burros at Lake Pleasant (*p* ≤ 0.001, Table 2). On the OTU scale, the diet diversity and dietary niche breadth of the burros were not significantly different between the two study areas (*p* ≥ 0.06, Table 2). 

**Figure 2:**
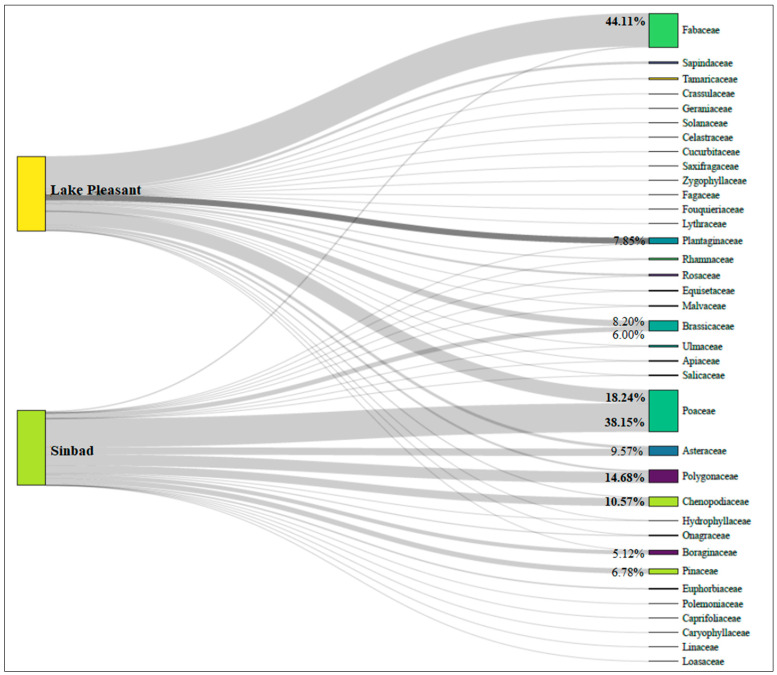
The relative read abundances of plant families in the summer diets of feral burros in Herd Management Areas at Lake Pleasant, Arizona, USA, and Sinbad, Utah, USA, identified from 50 fecal samples collected per study area in 2019. Percentages represent the most abundant plant family in the summer diet of each population (percentages > 10% are in bold).

At Lake Pleasant, we identified 29 families, 56 genera, and 72 species of plants in the burro fecal samples (Figure 2, Table 3 and Table A1). The most abundant family in the summer diet of burros was *Fabaceae*, with 44.11% of total read abundance, followed by *Poaceae* (18.24%) and *Brassicaceae* (8.19%) (Figure 2). *Parkinsonia florida* (20.09%), *Prosopis glandulosa* (18.24%), and *Lepidium lasiocarpum* (8.08%) were the three most prevalent species in the diet of the burros within this area. At Sinbad, the burro summer diet contained 24 families, 52 genera, and 65 species (Figure 2, Table 3 and Table A1); however, in contrast to Lake Pleasant, it comprised mainly *Poaceae* (38.15% of total read abundance), *Polygonaceae* (14.68%), and *Chenopodiaceae* (10.56% Figure 2). In Sinbad, the most abundant species in the summer diet of the burros were *Hesperostipa comata* (22.69%), *Eriogonum ovalifolium* (9.34%), and *Lepidium montanum* (4.89%).

At Lake Pleasant, we found 46.42% woody plants, 25.98% forbs, and 18.24% graminoids in the burros’ summer diet, whereas at Sinbad, we identified only 9.23% woody plants, 42.93% forbs, 38.15% graminoids, and 0.11% other plant forms in the diet of the burros. We could not identify 9.35% and 9.57% of the total read abundances with any plant form at Lake Pleasant and Sinbad, respectively.

## 4. Discussion

Few studies have examined the diet of burros, with most studies dating from the 1970s and having used microhistology (as reviewed in [40]). Subsequent studies have shown that microhistology tends to under-estimate proportions of forbs in the diets of herbivores because these plants tend to be more completely digested [21,22]. It is therefore possible that previous studies present an incomplete view of the burro diet. Although we only examined the burros’ diets over one month, using plant DNA barcoding, we provide a comprehensive and updated view of the summer diets of burros in two different ecosystems at the same point in time. The biggest limitation of our study was the short time period of fecal sample collection in both study areas, such that the sample collection does not represent all the species consumed by burros in a year. In the Sonoran Desert, burros are thought to consume jojoba year-round (J. Hall, BLM, written communication, May 2023), yet we did not identify this species in our samples. The samples from the Sonoran Desert were collected primarily near the lake, so although jojoba is thought to be present throughout the study area, the sampling locations may have influenced the results in that study area. 

Burros are highly adaptable. They are found in a variety of ecosystems from tropical islands to deserts [11], responding to different habitats with changes in their social organization [61] and diet [31]. Previous studies of burro diet in the United States have only been conducted in the desert habitats of California and Arizona because those are the areas in which burros are the most numerous, with no previous studies examining burro diets in less arid habitats such as Utah. Our results indicate that the burros in the juniper shrubland of Utah eat more grasses and consume less browse than what is found in the average summer diets of burros across several other populations (Table 1 and references therein). The proportions of forbs we found in the summer diets of the burros from both ecosystems are more comparable to spring diets reported from California and Arizona [16,28,29] than other summer diets in the United States. While microhistology is known to underestimate forbs present in the diet and plant DNA metabarcoding may overestimate it [62], other studies have also found that forbs are important for burros [40], especially in spring and summer [16].

There was limited overlap between our results from the Sonoran Desert and the diets of burros in the Mojave Desert [38] except for one study along the Colorado river in California [28] in which the results were comparable. In that study [28], researchers found that four plant species made up more than 50% of the burros’ annual diet, three of which were among the most common species found in our Sonoran Desert summer diet (*Parkinsonia florida*, *Plantago ovata*, and *Prosopis glandulosa*). Similarities between the Sonoran and Mojave Desert diets are likely most due to similar ecozones; both are subtropical desert ecosystems with some similar vegetation communities. In both systems, burros were reported to consume these highly digestible, nutritious species at higher proportions than other plant species. Burros may also be coupling foraging behavior with thermoregulation from shade trees during the hottest months in these systems [28]. Additionally, beans of *Parkinsonia* spp. and *Prosopis* spp. play major roles in burro diet when they are available, including in June and July (J. Hall, BLM, written communication, May 2023). Notably, in our diet results from the Sonoran Desert, there was a lack of cactus species. Burros have been reported to eat cactus [16,40,63], and there was evidence of their herbivory on cacti at Lake Pleasant (pers. obs. by the authors). It is possible that during our sampling period (June–July), there were sufficient alternative vegetation and moisture from forbs that it was not necessary for the burros to consume cacti. 

Our diversity index and dietary niche breadth results from the burros can be explained by the distinct ecosystems they inhabited. Tropical and subtropical ecosystems such as the Sonoran Desert have higher temporal and seasonal stability and abundance of flora and fauna than temperate ecosystems such as juniper shrublands [64] due to the historically constant and less severe temperature and climate fluctuations of the tropics. The primary forage for the burros in the Sonoran Desert during our study period was *Fabaceae* (44% of the diet), which includes mesquite, a highly digestible legume that is high in protein content, and the second-most represented family was *Poaceae* (18%) which includes grass species that are also highly nutritious. Together, these two plant families made up 65% of the burros’ summer diet in the Sonoran Desert. In Sinbad, *Poaceae* was first (38%), with three other species making up the next tier of the burros’ diet (*Polygonaceae* 14%, *Chenopodiaceae* 10%, and *Asteraceae* 9%) in June and July. The burros in Sinbad had a wider dietary niche breadth and higher diet diversity index because they had to rely on a wider variety of forage species to meet their nutrient needs. The burros in the temperate ecosystem also had greater temporal variability in the resources available to them [42]. Unlike the Sinbad burros, Lake Pleasant burros were able to rely on fewer plant species that had high levels of availability and nutrient value to meet the burros’ forage needs.

In a similar habitat to the Utah juniper shrubland ecosystem of our Sinbad site, and also using a plant DNA metabarcoding analysis of fecal samples, feral horse diets were found to be 69% graminoids and 19% forbs [22] compared to 38% graminoids and 43% forbs in the Sinbad burro diets. Interestingly, both horses and burros had similar proportions of woody plants in their summer diets (12% shrubs for horses and 9% for burros). Despite their reputation as browsers [15], in the Utah juniper shrubland system, burros appear to make up a large proportion of their diet with grasses and forbs, similar to more mesic habitats in Europe and India [32,33] and even along the Colorado River in the base of the Grand Canyon [30,31]. Feral burros are the domesticated descendants of African wild asses, which have been reported to rely on grass in both dry and wet seasons [65]. However, based on mesowear signatures, individual molar cusp shapes, and relief scores [66], African wild asses are also thought to be mixed feeders. The major component of the burros’ diet in the Sonoran Desert was a leguminous tree (*Prosopis glandulosa*, mesquite) which is highly digestible and has a higher protein content than grasses [67]. The fact that the burros in our study relied on woody browse in one ecosystem and forbs and graminoids in another demonstrates their ability to subsist on a variety of vegetation plant forms. 

The burros in the Sonoran Desert relied largely on *Prosopis* spp. In many parts of Africa and India, *Prosopis* is a rapidly spreading, non-native invasive species that is of concern to biodiversity, ecosystem services, and pastoralists due to its impact on native herbaceous plants [68]. In India, *Prosopis* is both helping and hindering the khur (*E. hemionus khur*), a subspecies of Asiatic wild ass endemic to the region that is categorized as Near Threatened with extinction [69]. While *Prosopis* forms part of the khur diet (they eat both the leaves and seed pods [70]), khur also contribute to its spread by dispersing germinable seeds in their dung, thus helping to establish this tree, which reduces the abundance of herbaceous species that khur also rely on [70,71]. Understanding that burros may fulfill a similar role to their native relatives is important to managers and scientists in the desert southwest of the United States. It is also valuable for managers in other parts of the world where *Prosopis* is invasive and domestic donkeys are common. 

## 5. Conclusions

Understanding animal diets can help ecologists assess the interactions of a species with its environment and the potential effects on other inhabitants of the ecosystem. We conclude that feral burros are highly adaptable and utilize forages with the highest nutritional value in whichever ecosystems they are found. Instead of being defined as strict browsers or grazers, they appear to be employing a mixed feeding strategy, similar to their ancestor, the African wild ass, to meet their nutritional needs across the varied ecosystems they inhabit.

## Figures and Tables

**Table 2:** Results of a comparison of dietary niche breadth (richness) and Shannon diversity index in 95% of total DNA sequence reads generated from burro fecal samples collected from 24 June to 16 July 2019 in Herd Management Areas at Lake Pleasant, Arizona, USA (50 fecal samples, 148 exact sequence variants, and 55 operational taxonomic units), and Sinbad, Utah, USA (50 fecal samples, 202 exact sequence variants, and 52 operational taxonomic units).

	Lake Pleasant (Mean ± SD)	Sinbad (Mean ± SD)	W	* p *
Dietary niche breadth (ESVs)	8.26 ± 3.14	11.46 ± 4.44	655	≤0.001
Dietary niche breadth (OTUs)	6.70 ± 2.13	7.82 ± 3.09	983	0.06
Shannon’s diversity (ESVs)	1.56 ± 0.55	1.94 ± 0.52	696	≤0.001
Shannon’s diversity (OTUs)	1.40 ± 0.47	1.57 ± 0.48	986	0.07

**Table 3:** Genera (with their corresponding families and species) composing > 1% of feral burro summer diets, identified using a DNA metabarcoding approach from 50 fecal samples per study area collected between 24 June and 16 July 2019. Data are based on 56 and 52 operational taxonomic units representing 95% of the total reads at the Lake Pleasant (Arizona, USA) and Sinbad (Utah, USA) Herd Management Areas (HMA), respectively. Where no species is given, the genera represent more than one species in the diet, and we provided percentages for the genus and for each species separately. Plant forms: F = forbs; G = graminoids; W = woody plants.

	Lake Pleasant HMA					Sinbad HMA			
Family	Genus	Species	Plant Form	% in Diet	Family	Genus	Species	Plant Form	% in Diet
*Fabaceae*	*Parkinsonia*			20.09	*Poaceae*	*Hesperostipa*	*Hesperostipa comata*	G	22.69
		*Parkinsonia florida*	W	19.98	*Polygonaceae*	*Eriogonum*			12.79
		*Parkinsonia microphylla*	W	0.11			*Erigonum alatum*	F	0.66
	*Prosopis*			18.47			*Erigonum bicolor*	F	0.89
		*Prosopis glandulosa*	W	18.24			*Eriogonum cernuum*	F	1.89
		*Prosopis juliflora*	W	0.23			*Eriogonum ovalifolium*	F	9.34
	Unknown			10.51		Unknown			12.23
*Brassicaceae*	*Lepidium*			8.20	*Brassicaceae*	*Lepidium*	*Lepidium montanum*	F	4.89
		*Lepidium lasiocarpum*	F	8.08	*Boraginaceae*	*Lappula*	*Lappula occidentalis*	F	4.67
		*Lepidium virginicum*	F	0.11	*Chenopodiaceae*	*Atriplex*	*Atriplex canescens*	W	4.34
*Plantaginaceae*	*Plantago*			7.85	*Asteraceae*	*Ambrosia*	*Ambrosia acanthicarpa*	F	3.89
		*Plantago ovata*	F	7.74	*Poaceae*	*Bouteloua*	*Bouteloua gracilis*	G	3.89
		*Plantago patagonica*	F	0.11		*Poa*			3.78
*Poaceae*	*Cynodon*	*Cynodon dactylon*	G	6.70			*Poa fendleriana*	G	0.67
	*Poa*			4.85			*Poa pratensis*	G	2.45
		*Poa annua*	G	3.93			*Poa secunda*	G	0.67
		*Poa bigelovii*	G	0.92	*Chenopodiaceae*	*Chenopodium*			2.56
*Fabaceae*	*Olneya*	*Olneya tesota*	W	2.77			*Chenopodium album*	F	0.11
*Polygonaceae*	*Eriogonum*			2.31			*Chenopodium fremontii*	F	2.45
		*Eriogonum capillare*	F	0.35	*Pinaceae*	*Pinus*			2.46
		*Eriogonum fasciculatum*	W	0.35			*Pinus discolor*	W	1.56
		*Eriogonum ovalifolium*	F	1.38			*Pinus edulis*	W	0.56
		*Eriogonum polycladon*	F	0.11			*Pinus monophylla*	W	0.33
		*Eriogonum thomasii*	F	0.11	*Chenopodiaceae*	*Salsola*	*Salsola tragus*	F	2.11
*Asteraceae*	*Helianthus*	*Helianthus annuus*	F	1.85	*Poaceae*	*Panicum*	*Panicum virgatum*	G	1.89
*Tamaricaceae*	*Tamarix*	*Tamarix chinensis*	W	1.73	*Asteraceae*	*Helianthus*	*Helianthus annuus*	F	1.69
*Poaceae*	*Bromus*			1.27	*Poaceae*	*Bromus*	*Bromus tectorum*	G	1.44
		*Bromus tectorum*	G	0.69	*Onagraceae*	*Oenothera*			1.33
		*Bromus hordeaceus*	G	0.46			*Oenothera caespitosa*	F	0.33
		*Bromus japonicus*	G	0.11			*Oenothera pallida*	F	1.00
	*Panicum*			1.27	*Asteraceae*	*Artemisia*			1.12
		*Panicum capillare*	G	0.92			*Artemisia dracunculus*	F	0.11
		*Panicum miliaceum*	G	0.23			*Artemisia frigida*	W	1.00
							*Artemisia tridentada*	W	0.01

## Data Availability

All data presented in this study are available in the manuscript, Appendix A, Table A1. Data generated in this study are available in a U.S. Geological Survey data release (Schoenecker et al., 2023, https://doi.org/10.5066/P9I5X8V7).

## Appendix A

**Table A1:** Genera (with their corresponding families and species) composing < 1% of feral burro diet identified using a DNA metabarcoding approach in 50 fecal samples per study area collected between 24 June and 16 July 2019. Data are based on 56 and 52 operational taxonomic units representing 95% of the total reads at the Lake Pleasant (Arizona, USA) and Sinbad (Utah, USA) Herd Management Areas (HMA), respectively. Where no species is given, the genera represent more than one species in the diet, and we provided percentages for the genus and for each species separately.

Lake Pleasant HMA				Sinbad HMA			
Family	Genus	Species	% in Diet	Family	Genus	Species	% in Diet
*Fabaceae*	*Lotus*	*Lotus humistratus*	0.92	*Poaceae*	*Achnatherum*		0.89
*Poaceae*	*Triticum*	*Triticum aestivum*	0.92			*Achnatherum aridum*	0.22
*Fabaceae*	*Acacia*	*Acacia greggii*	0.80			*Achnatherum nelsonii*	0.67
*Chenopodiaceae*	*Chenopodium*	*Chenopodium murale*	0.80			*Achnatherum thurberianum*	<0.01
*Malvaceae*	*Malva*	*Malva parviflora*	0.69	*Chenopodiaceae*	*Bassia*	*Bassia hyssopifolia*	0.89
*Asteraceae*	*Artemisia*	*Artemisia campestris*	0.58	*Polygonaceae*	*Polygonum*	*Polygonum aviculare*	0.89
*Poaceae*	*Schismus*	*Schismus arabicus*	0.58	*Poaceae*	*Sporobulus*	*Sporobolus flexuosus*	0.78
*Malvaceae*	*Sphaeralcea*	*Sphaeralcea coulteri*	0.58	*Brassicaceae*	*Chorispora*	*Chorispora tenella*	0.67
*Poaceae*	*Echinochloa*	*Echinochloa colona*	0.46	*Fabaceae*	*Hedysarum*	*Hedysarum boreale*	0.67
*Asteraceae*	*Gutierrezia*	*Gutierrezia sarothrae*	0.46	*Asteraceae*	*Packera*	*Packera multilobata*	0.67
*Equisetaceae*	*Equisetum*	*Equisetum laevigatum*	0.35	*Hydrophyllaceae*	*Phacelia*	*Phacelia constancei*	0.67
*Geraniaceae*	*Erodium*	*Erodium cicutarium*	0.35	*Ulmaceae*	*Ulmus*	*Ulmus pumila*	0.67
*Poaceae*	*Hilaria*		0.35	*Asteraceae*	*Chaenactis*	*Chaenactis douglasii*	0.56
		*Hilaria belangeri*	0.12	*Asteraceae*	*Cirsium*	*Cirsium undulatum*	0.44
		*Hilaria mutica*	0.12	*Boraginaceae*	*Cryptantha*	*Cryptantha johnstonii*	0.44
		*Hilaria rigida*	0.11	*Brassicaceae*	*Descurainia*	*Descurainia pinnata*	0.44
*Solanaceae*	*Lycium*	*Lycium fremontii*	0.35	*Chenopodiaceae*	*Halogeton*	*Halogeton glomeratus*	0.44
*Boraginaceae*	*Amsinckia*	*Amsinckia tessellata*	0.23	*Malvaceae*	*Sphaeralcea*	*Sphaeralcea coccinea*	0.44
*Poaceae*	*Aristida*	*Aristida adscensionis*	0.23	*Asteraceae*	*Gutierrezia*	*Gutierrezia sarothrae*	0.33
*Celastraceae*	*Canotia*	*Canotia holacantha*	0.23	*Poaceae*	*Elymus*	*Elymus trachycaulus*	0.22
*Rosaceae*	*Cercocarpus*	*Cercocarpus montanus*	0.23	*Asteraceae*	*Erigeron*	*Erigeron compositus*	0.22
*Saxifragaceae*	*Heuchera*	*Heuchera eastwoodiae*	0.23	*Rhamnaceae*	*Frangula*	*Frangula betulifolia*	0.22
*Fabaceae*	*Hoffmannseggia*	*Hoffmannseggia glauca*	0.23	*Asteraceae*	*Machaeranthera*	*Machaeranthera gracilis*	0.22
*Zygophyllaceae*	*Larrea*	*Larrea tridentata*	0.23	*Plantaginaceae*	*Plantago*	*Plantago patagonica*	0.22
*Rosaceae*	*Prunus*		0.23	*Asteraceae*	*Tragopogon*	*Tragopogon dubius*	0.22
		*Prunus fasciculata*	0.11	*Apiaceae*	*Cymopterus*	*Cymopterus acaulis*	0.11
		*Prunus serotina*	0.11	*Equisetaceae*	*Equisetum*	*Equisetum laevigatum*	0.11
*Asteraceae*	*Pseudognaphalium*	*Pseudognaphalium luteoalbum*	0.23		*Ipomopsis*	*Ipomopsis congesta*	0.11
*Poaceae*	*Cenchrus*	*Cenchrus longispinus*	0.11	*Polemoniaceae*	*Linanthus*	*Linanthus pungens*	0.11
*Asteraceae*	*Conyza*	*Conyza bonariensis*	0.11	*Linaceae*	*Linum*	*Linum tenuifolium*	0.11
*Apiaceae*	*Daucus*	*Daucus pusillus*	0.11	*Loasaceae*	*Mentzelia*	*Mentzelia montana*	0.11
*Poaceae*	*Distichlis*	*Distichlis spicata*	0.11	*Caryophyllaceae*	*Paronychia*	*Paronychia sessiliflora*	0.11
*Poaceae*	*Festuca*	*Festuca octoflora*	0.11	*Rosaceae*	*Purshia*	*Purshia tridentata*	0.11
*Fouquieriaceae*	*Fouquieria*	*Fouquieria splendens*	0.11	*Salicaceae*	*Salix*	*Salix exigua*	0.11
*Lythraceae*	*Lythrum*	*Lythrum californicum*	0.11	*Onagraceae*	*Epilobium*	*Epilobium brachycarpum*	<0.01
*Fabaceae*	*Medicago*	*Medicago polymorpha*	0.11	*Euphorbiaceae*	*Euphorbia*	*Euphorbia brachycera*	<0.01
*Hydrophyllaceae*	*Phacelia*	*Phacelia distans*	0.11	*Asteraceae*	*Gaillardia*	*Gaillardia spathulata*	<0.01
*Salicaceae*	*Populus*	*Populus fremontii*	0.11	*Asteraceae*	*Tanacetum*	*Tanacetum vulgare*	<0.01
*Rosaceae*	*Purshia*	*Purshia stansburiana*	0.11	*Asteraceae*	*Tetradymia*	*Tetradymia spinosa*	<0.01
*Fagaceae*	*Quercus*	*Quercus turbinella*	0.11				
*Fabaceae*	*Senna*	*Senna covesii*	0.11				
*Asteraceae*	*Sonchus*	*Sonchus oleraceus*	0.11				
*Poaceae*	*Sporobolus*	*Sporobolus cryptandrus*	0.11				
*Asteraceae*	*Xanthium*	*Xanthium strumarium*	0.11				
*Asteraceae*	*Ambrosia*	*Ambrosia artemisiifolia*	<0.01				
*Poaceae*	*Bouteloua*	*Bouteloua aristidoides*	<0.01				
*Fabaceae*	*Dalea*	*Dalea mollis*	<0.01				
*Poaceae*	*Paspalum*	*Paspalum dilatatum*	<0.01				
*Solanaceae*	*Physalis*	*Physalis angulata*	<0.01				
